# Survival of direct composite restorations placed under general anesthesia in adult patients with intellectual and/or physical disabilities

**DOI:** 10.1007/s00784-020-03770-y

**Published:** 2021-01-15

**Authors:** Mona Shaghayegh Maes, Philipp Kanzow, Valentina Hrasky, Annette Wiegand

**Affiliations:** Department of Preventive Dentistry, Periodontology and Cariology, University Medical Center Göttingen, Robert-Koch-Str. 40, D-37075 Göttingen, Germany

**Keywords:** Composite, Restoration, Longevity, Disability, Kaplan-Meier statistics

## Abstract

**Objectives:**

This study aimed to assess the survival of direct composite restorations placed under general anesthesia in adult patients with intellectual and/or physical disabilities.

**Materials and methods:**

Survival of composite restorations placed under general anesthesia in adult patients with intellectual and/or physical disabilities was retrospectively analyzed. Failure was defined as the need for replacement of at least one surface of the original restoration or extraction of the tooth. Individual-, tooth-, and restoration-related factors were obtained from dental records. Five-year mean annual failure rate (mAFR) and median survival time were calculated (Kaplan-Meier statistics). The effect of potential risk factors on failure was tested using univariate log-rank tests and multivariate Cox-regression analysis (*α* = 5%).

**Results:**

A total of 728 restorations in 101 patients were included in the analysis. The survival after 5 years amounted to 67.7% (5-year mAFR: 7.5%) and median survival time to 7.9 years. Results of the multivariate Cox-regression analysis revealed physical disability (HR: 50.932, *p* = 0.001) and combined intellectual/physical disability (HR: 3.145, *p* = 0.016) compared with intellectual disability only, presence of a removable partial denture (HR: 3.013, p < 0.001), and restorations in incisors (HR: 2.281, *p* = 0.013) or molars (HR: 1.693, *p* = 0.017) compared with premolars to increase the risk for failure.

**Conclusion:**

Composite restorations placed under general anesthesia in adult patients with intellectual and/or physical disabilities showed a reasonable longevity as 67.7% survived at least 5 years.

**Clinical relevance:**

Survival of composite restorations depends on risk factors that need to be considered when planning restorative treatment in patients with intellectual and/or physical disabilities. NCT04407520

## Introduction

Dental composites have been becoming the materials of choice for direct restorations in permanent teeth, especially when considering the phase down of amalgam. Recent systematic reviews and meta-analyses reported mean annual failure rates of 0 to 4% for anterior [1] and 0.6 to 4.2% for posterior [2] composite restorations. However, more recent studies rather focus on the survival of composite restorations, but particularly on material-, tooth-, and patient-related factors that might affect survival [3, 4]. With regard to patient-related factors, caries risk and related variables were shown to significantly affect the survival of composite restorations [3, 4].

Patients with special needs, such as elderly people or persons with intellectual and/or physical disability, often belong to the group of patients with high caries risk. Interestingly, only few data on the performance of direct restorations in this specific group of patients have been published. In elderly and geriatric patients, median survival of composite restorations ranged from 5.5 to 9.9 years [5, 6]. Tong et al. [7] reported the 5-year survival of composite restorations in frail older adults to amount to 60.5%. In children and adults with intellectual and/or physical disability, the 5-year survival of single- and multiple-surface composite restorations amounted to 100% and 66.9%, respectively. Composite restorations placed under general anesthesia showed a better survival than restorations placed conventionally [8]. In another study, 77.3% of composite restorations placed under general anesthesia in children and adults with special needs survived after 2 years [9].

As data on the survival of composite restorations in adult patients with intellectual and/or physical disabilities are very limited, this retrospective study aimed to assess the survival of direct composite restorations of adult patients with intellectual and/or physical disabilities placed under general anesthesia. Additionally, we evaluated individual-, tooth-, and restoration-related risk factors on restorations’ longevity.

## Materials and methods

This study was approved by the ethics committee of the University Medical Center Göttingen (no. 15/1/18) and registered on ClinicalTrials.gov (NCT04407520). Data were collected from digital and paper-based dental records of adult patients with intellectual and/or physical disabilities that were treated under general anesthesia in the Department of Preventive Dentistry, Periodontology and Cariology. The following inclusion criteria were defined: direct anterior and/or posterior composite restoration placed in general anesthesia in permanent teeth of adult patients with intellectual and/or physical disability, general anesthesia performed between January 2011 and December 2019, restoration made from a nano-hybrid composite placed in etch&rinse technique without rubber dam. Patients aged below 18 years, restorations on root canal-treated teeth, and restorations without a minimum follow-up of 14 days were excluded from the analysis.

One investigator (M.M.) reviewed all records and obtained the following data: date of the placement of the original anterior and/or posterior composite restoration and date of first re-intervention (re-restoration of the same tooth including at least one surface of the original restoration or tooth extraction) or date of the last checkup of the patient. Further individual and tooth-/restoration-related variables were assessed: type of disability (intellectual/physical/both), living situation (care facility/private setting), oral hygiene (alone/with support/impossible), nutrition (without restrictions/pureed or liquid food/feeding tube), presence of a removable partial denture (yes/no), postoperative checkup within 3 months (yes/no), tooth location (upper/lower jaw), tooth type (anterior/premolar/molar), load-bearing restoration (yes/no), number of surfaces (1/2/≥ 3), gender, age, average number of follow-up visits per year, number of decayed teeth, number of missing teeth, number of filled teeth, and DMFT score.

For all variables except follow-up visits per year, the status prior to the treatment session of initial restoration was evaluated. Restorations including the occlusal or incisal surface were defined as “load-bearing”. The average number of follow-up visits per year was only calculated for follow-up intervals > 6 months to exclude unreliable results in case of short follow-up intervals.

### Outcome

All restorations without any further interventions until the date of last checkup were considered as survived. Composite restorations were rated as failed if at least one of the involved surfaces was re-restored or the tooth was extracted. If in case of re-intervention, restorations were regarded as failed at the date of intervention. Censoring at the time of intervention was performed if endodontic treatments on the original restored tooth became necessary. Censoring was also performed in case a mesial-occlusal (mo) or distal-occlusal (od) restoration was placed during follow-up of an initial od or mo restoration, respectively, as a clear distinction between two separate restorations or a combined restoration was not possible.

### Statistical analysis

For calculating the time-until-event or time-until-censoring (years) of direct composite restorations, Microsoft Excel for Mac (version 16.33) was used.

Statistical analysis was performed using the software R (version 3.6.2, www.r-project.org) and the packages “survminer” (version 0.4.6), “survival” (version 3-1.11), and “dplyr” (version 0.8.5). The level of significance was set at *α* = 0.05. Longevity of restorations was assessed up to 8 years by Kaplan-Meier statistics. Mean annual failure rate (mAFR) at 5 years was calculated by the following formula [10].

$$ {\displaystyle \begin{array}{c}{\left(1-y\right)}^5=1-x\\ {}y=1-\sqrt[5]{1-x}\end{array}} $$

*y* = 5-year mean annual failure rate; *x* = failure rate

Log-rank tests (categorical variables) and Cox regression (continuous variables) were used to assess the univariate effect of both individual-, tooth-, and restoration-related variables. Subsequently, variables with a significant effect were used in a multi-variate Cox regression model with shared frailty of correlated observations (restorations within the same patient). Hazard ratios (HR) and their respective 95% confidence intervals were calculated for factors associated with failure.

## Results

A total of 1275 direct composite restorations were placed in 185 patients from January 2011 to December 2019. A total of 547 restorations had to be excluded due to previous root canal treatment (*n* = 6) or follow-up of less than 14 days (*n* = 541). Thus, 728 restorations in 101 patients (mean age: 37.3 ± 13.1) were included in the analysis.

**Fig. 1:**
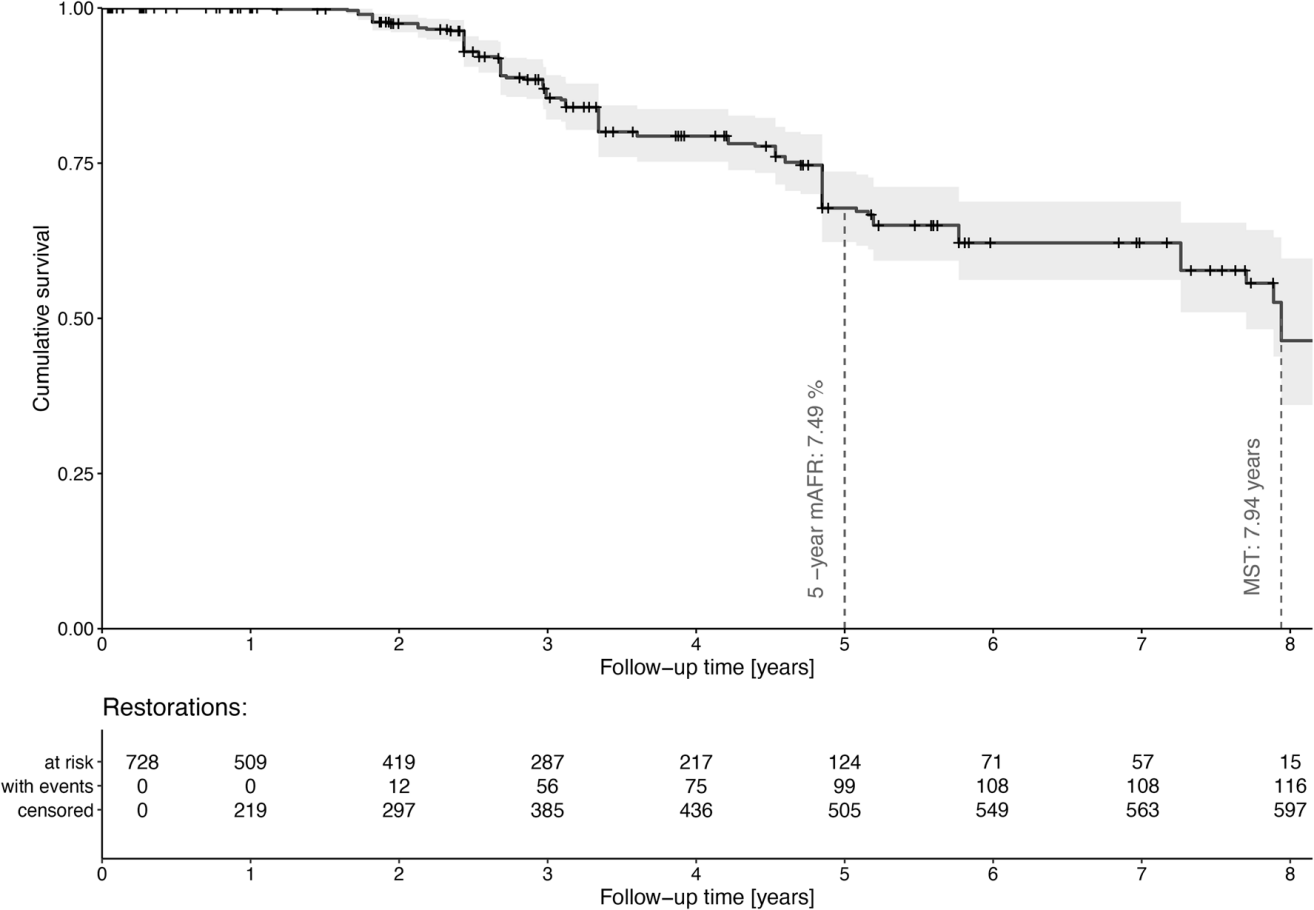
Kaplan-Meier survival curve with 95% confidence interval (over 8 years) of placed restorations, number of restorations at risk, restorations with events and censored restorations as well as mean annual failure rate (mAFR) after 5 years, and median survival time (MST)

The follow-up time amounted to 2.9 ± 2.3 (min: 14 days, max: 8.7 years), 116 restorations failed (re-restoration/replacement: *n* = 57, extraction: *n* = 59). Longevity of restorations was assessed up to 8 years by Kaplan-Meier statistics. The survival after 5 years amounted to 67.7% (5-year mAFR: 7.5%) and median survival time to 7.9 years (Fig. 1).

Tables 1 and 2 present potential risk factors that were subjected to the univariate analysis. Type of disability, nutrition, presence of a removable partial denture, postoperative checkup within 3 months, tooth type, load-bearing restorations (Table 1), age, decayed teeth, missing teeth, and DMFT value (Table 2) were found to be significant with regard to failure.

**Table 1:** Characteristics of patients and involved restorations and p-values of the univariate analysis by log-rank tests (categorial variables)

	Number of restorations		% of restorations		*p* value
	Total (*N* = 728)	Failed (*N* = 116)	Total (*N* = 728)	Failed (*N* = 116)	
Type of disability					< 0.001
Intellectual (*n* = 41)	339	27	46.6	23.3	
Physical (*n* = 7)	79	30	10.9	25.9	
Both intellectual and physical (*n* = 53)	310	59	42.6	50.9	
Gender					n.s.
Male (*n* = 51)	398	80	54.7	69.0	
Female (*n* = 50)	330	36	45.3	31.0	
Living situation					n.s.
Care facility (*n* = 52)	325	53	44.6	45.7	
Private setting (with family or alone, *n* = 46)	380	60	52.2	51.7	
Unknown (*n* = 3)	23	3	3.2	2.6	
Oral hygiene					n.s.
Alone (*n* = 37)	282	42	38.7	36.2	
With support (*n* = 48)	333	57	45.7	49.1	
Impossible (*n* = 12)	76	10	10.4	8.6	
Unknown (*n* = 4)	37	7	5.1	6.0	
Nutrition					< 0.001
Without restrictions (*n* = 72)	517	57	71.0	49.1	
Pureed/liquid food (*n* = 17)	120	32	16.5	27.6	
Feeding tube (*n* = 9)	56	20	7.7	17.2	
Unknown (*n* = 3)	35	7	4.8	6.0	
Removable partial denture					< 0.001
Yes (*n* = 17)	141	44	19.4	37.9	
No (*n* = 84)	587	72	80.6	62.1	
Postoperative checkup within 3 months					0.038
Yes	278	13	38.2	11.2	
No	450	103	61.8	88.8	
Number of surfaces of the restoration					n.s.
1	365	54	50.1	46.6	
2	201	25	27.6	21.6	
≥ 3	162	37	22.3	31.9	
Tooth type					< 0.001
Anterior	257	58	35.3	50.0	
Premolar	181	21	24.9	18.1	
Molar	290	37	39.8	31.9	
Load-bearing restoration^a^					< 0.001
Yes	426	56	58.5	48.3	
No	302	60	41.5	51.7	
Location					n.s.
Upper jaw	410	74	56.3	63.8	
Lower jaw	318	42	43.7	36.2	

Due to the effect of rounding, some numbers do not sum up to 100%

*n* number of patients, *n.s.* not significant

^a^Restorations including occlusal or incisal surfaces

**Table 2:** Characteristics of patients and involved restorations and *p* values of univariate Cox regressions (continuous variables)

Parameter	Mean ± SD	*p* value
Age^a^ (years)	37.3 ± 13.1	0.011
Decayed teeth^b^	12.5 ± 6.6	< 0.001
Missing teeth^b^	5.4 ± 4.3	0.011
Filled teeth^b^	3.7 ± 3.9	n.s.
DMFT-score^b^	21.6 ± 7.4	< 0.001
Average number of follow-up visits per year	1.5 ± 1.3	n.s.

*n.s.* not significant

^a^At time of initial restoration

^b^Prior to treatment session of initial restoration

Results of the multivariate Cox regression analysis revealed physical disability and combined intellectual/physical disability compared with intellectual disability only and the presence of a removable partial denture to increase the risk for failure. Furthermore, restorations in incisors (1 surface: 47.1%, two surfaces: 20.6%, ≥ 3 surfaces: 32.3%) or molars (1 surface: 55.5%, 2 surfaces: 29.0 %, ≥ 3 surfaces: 15.5%) were at higher risk for failure compared with premolars (1 surface: 45.9%, 2 surfaces: 35.4%, ≥ 3 surfaces: 18.8%, Table 3).

**Table 3:** Parameters in the multivariate Cox regression analysis (*p* value, hazard ratio, 95% CI = 95% confidence)

Parameter	*p* value	Hazard ratio (95% CI)
Type of disability		
Physical vs. intellectual (= 1)	*0.001*	HR = 50.932 (4.72–549.86)
Physical vs. both intellectual and physical (= 1)	*0.003*	HR = 16.197 (2.54–103.20)
Both intellectual and physical vs. intellectual (= 1)	*0.016*	HR = 3.145 (1.24–7.99)
Nutrition		
Pureed/liquid food vs. without restrictions (= 1)	0.480	
Pureed/liquid food vs. feeding tube (= 1)	0.087	
Feeding tube vs. without restrictions (= 1)	0.468	
Removable partial denture		
Yes vs. no (= 1)	*< 0.001*	HR = 3.013 (1.61–5.64)
Postoperative checkup within 3 months		
Yes vs. no (= 1)	0.691	
Tooth type		
Incisor vs. premolar (= 1)	*0.013*	HR = 2.281 (1.19–4.39)
Molar vs. incisor (= 1)	0.402	
Molar vs. premolar (= 1)	*0.017*	HR = 1.693 (1.10–2.61)
Load bearing restoration		
Yes vs. no (= 1)	0.182	
Age	0.669	
Decayed teeth	0.175	
Missing teeth	0.232	
DMFT score	0.798	

Significant *p*-values are printed in italics

**Fig. 2:**
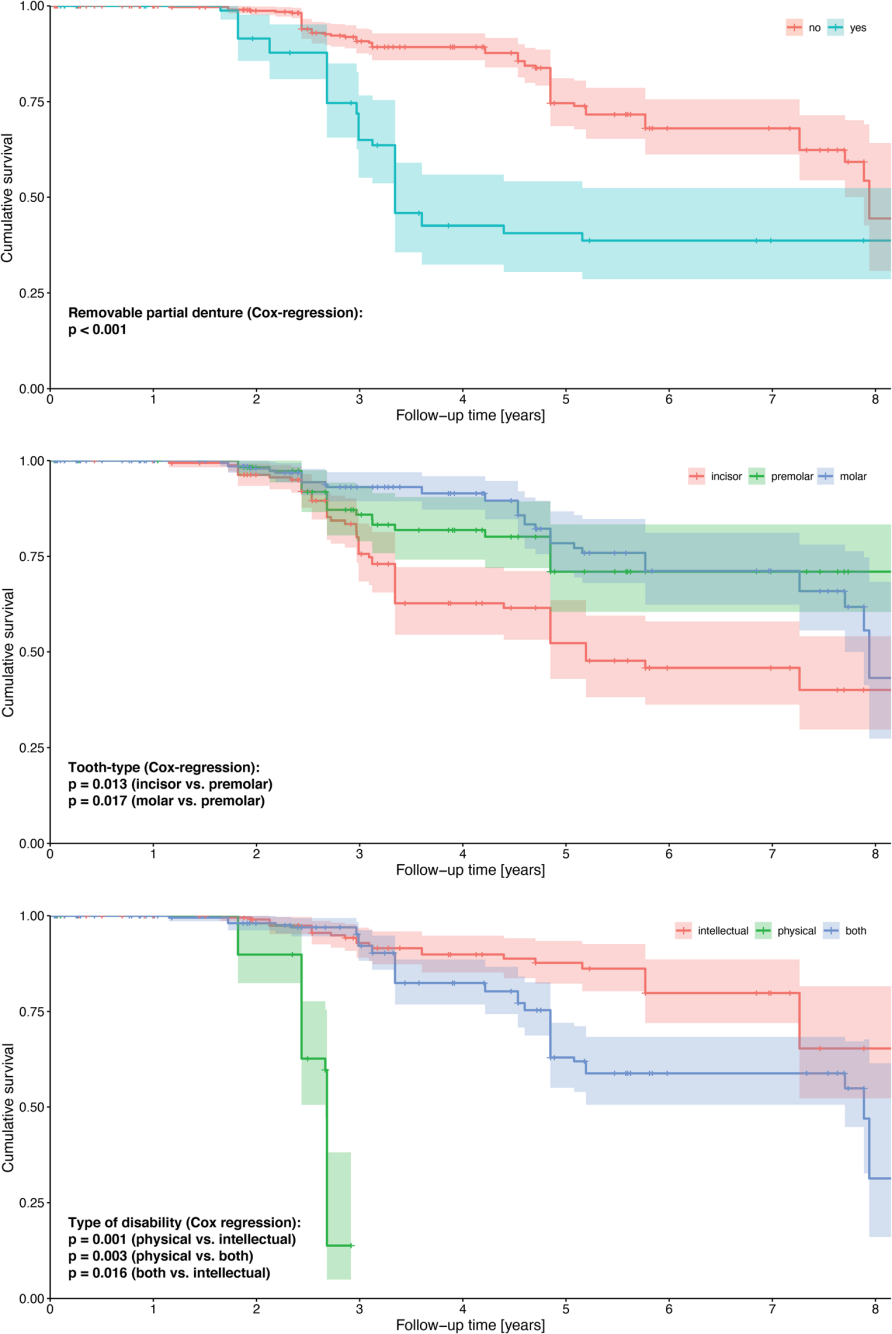
Kaplan-Meier survival graphs and respective 95% confidence intervals (over 8 years) of categorical variables being significant in multi-variate Cox regression analysis with shared frailty

Kaplan-Meier survival graphs of categorical variables being significant are shown in Fig. 2.

## Discussion

Placement of direct restorations is the most common procedure when patients with intellectual disability are treated under general anesthesia [11, 12]. However, conflicting data on the use of specific materials exist: while some authors report the frequent use of composites for treatment of adult special needs patients [9, 12, 13], others refuse the use of composite for restoring posterior teeth not least as data on the longevity are scarce [14].

This study reported the median survival time of composite restorations in adult patients with disabilities to amount to 7.9 years and the 5-year survival to 67.7%. Longevity of composite restorations is therefore in the range of other studies reporting on composite restorations in different groups of special needs patients [5–7].

This is the first study that analyzed the longevity of composite restorations solely in adult patients with intellectual and/or physical disabilities. However, validity is limited by the fact that a very diverse group of patients with various congenital, acquired, and neurodegenerative disorders was included and no standardization with regard to the degree of disability was possible. On the other hand, all patients were united by the fact that dental treatment was only possible under general anesthesia. The vast majority of treatments (about 95%) was performed by the same operator. Physically disabled patients showed a higher risk for failure than patients with intellectual or both intellectual/physical disabilities. This result has to be interpreted with great caution, as comparatively few patients were affected from physical disability only (Table 1 and Fig. 2). However, combined physical/intellectual disability increased the risk for restoration failure, potentially as caries risk is further increased, e.g., due to limitations in oral hygiene and/or nutrition.

Caries experience (DMFT) of our patients was distinctly higher compared with adult athletes with intellectual disabilities [15, 16] and even to adults with intellectual disabilities working in special day-care institutions in Germany [17]. Consequently, the number of decayed and missing teeth, the DMFT score, and the presence of removable partial dentures were found to be significant with respect to restoration failure in the univariate analyses. However, in the multivariate analysis, only the presence of removable partial dentures remained significant.

Finally, the tooth type had a significant effect on restoration failure, as premolars showed a significantly lower risk than molars and incisors. This result is in line with previous studies on composite longevity in patients without disability [18, 19]. In this study, anterior teeth presented more multi-surface restorations compared with premolars and molars, probably contributing to the higher risk of failure. Moreover, patients with special needs are at higher risk for dental trauma [20], which might also affect longevity of anterior restorations.

Other restoration-related parameters, such as load-bearing restoration and number of involved surfaces, were significant only in the univariate model, but not in the multivariate analysis; probably, these parameters do not precisely account for restoration size and depth.

Due to the retrospective design, this study presents some methodological limitations: data were extracted from digital and paper-based records, so that only variables that were consistently documented could be obtained. Potentially, restorations might have been repaired or replaced outside our department and without our knowledge. However, this is overall unlikely, as our department is the only clinic in the near surrounding offering dental treatment (usually necessary in general anesthesia) for patients with severe disabilities.

Despite a large number of restorations was included in the statistical analysis, the overall number of patients was limited. However, statistical analysis controlled for multiple restorations of the same patient. Unfortunately, a high number of restorations (541 out of 1275) had to be excluded from the analysis as no follow-up was available. As in other retrospective studies dealing with restoration survival in special needs patients [5, 7], the overall censoring rate was high resulting in a relatively low number of restorations that could be followed up beyond 5 years. This aspect might affect the Cox regression analysis, especially regarding the tooth type, as differences between molars and premolars became only evident at the end of the observation period.

Due to the severe impairment, patients were often unable to attend the postoperative check-up or routine dental recall appointments after treatment in general anesthesia. For these patients, survival of restorations might be reduced as they are missing preventive treatment, like oral hygiene instruction or fluoridation, reducing the risk for (secondary) caries.

For patients included in the analysis, restoration survival was affected by the attendance of the postoperative check-up. Notably, these patients did not attend recall appointments on a regular basis so that the number or frequency of recall appointments could not be considered in the statistical analysis. Alternatively, the postoperative check-up was considered as variable. Nonetheless, the average number of patients attending the postoperative check-up was low, probably also due to the effect that dental visits are often difficult for patients with severe disabilities.

In conclusion, composite restorations placed under general anesthesia in adult patients with intellectual and/or physical disabilities showed reasonable survival of at least 67.7% after 5 years. Further studies are needed comparing composite restorations to other direct fillings.
